# CYMP-AS1 Promotes Ovarian Cancer Progression by Enhancing the Intracellular Translocation of hnRNPM and Reducing the Stability of AXIN2 mRNA

**DOI:** 10.32604/or.2025.064367

**Published:** 2025-07-18

**Authors:** Yuhan Wang, Yimei Meng, Wanqiu Xia, Yusen Liang, Yaru Wang, Peiling Li, Lei Fang

**Affiliations:** Department of Obstetrics and Gynecology, The Second Affiliated Hospital of Harbin Medical University, Harbin, 150001, China

**Keywords:** CYMP antisense RNA 1 (CYMP-AS1), heterogeneous nuclear ribonucleoprotein M (hnRNPM), mRNA stability, ovarian cancer (OC), long non-coding RNAs (lncRNAs)

## Abstract

**Background:**

Ovarian cancer (OC) is a representative malignancy of the female reproductive system, with a poor prognosis. Long non-coding RNAs (lncRNAs) crucially affect tumor development. This study aimed to identify lncRNAs that potentially participated in OC.

**Methods:**

LncRNA expression in cells and tissues was quantified using reverse transcription-quantitative PCR, while fluorescence *in situ* hybridization determined their cellular localization. Various *in vitro* assays, together with a mouse xenograft model, were employed to elucidate the function of CYMP antisense RNA 1 (CYMP-AS1) in OC. The molecular mechanisms underlying CYMP-AS1 regulation were investigated through RNA pull-down and immunoprecipitation assays, immunofluorescence staining, western blotting, and mRNA stability assays.

**Results:**

This study identified a previously unreported lncRNA, CYMP-AS1, which exhibits increased expression in the cytoplasm of OC tissues and cells. Knockout of CYMP-AS1 reduced the OC cell proliferation, migration, invasion, and epithelial-mesenchymal transition (EMT). CYMP-AS1 directly interacts with heterogeneous nuclear ribonucleoprotein M (hnRNPM), inducing its intracellular translocation and reducing the stability of Axis inhibition protein 2 (AXIN2) mRNA. This process ultimately elevated the expression of Wnt/β-catenin signaling pathway-related proteins.

**Conclusion:**

This study confirms CYMP-AS1 as a novel biomarker in OC progression and suggests that the CYMP-AS1/hnRNPM/AXIN2 axis may offer an innovative strategy for OC treatment.

## 1 Introduction

Ovarian cancer (OC) ranks seventh among all cancer types for females and is the leading cause of gynecological tumor-related death [1–3]. Despite the availability of multiple treatment modalities, prognosis is still poor, and the 5-year survival rate reaches 30%–47% [4,5]. This high mortality is primarily attributed to cancer metastasis from the ovaries to adjacent organs or the abdominal cavity [6], a process that involves multiple steps and factors [7,8]. Consequently, comprehending relevant molecular mechanisms can well assist in identifying effective diagnostic and therapeutic targets for patients with OC.

Long non-coding RNAs (lncRNAs) are RNA molecules with a length of over 200 nucleotides and possess limited or no protein-coding capacity [9,10]. According to existing research, these molecules participate in cell migration, proliferation, apoptosis, invasion, epithelial-to-mesenchymal transition (EMT) progression and other pathophysiological processes [11–13]. Recent investigations have also elucidated the significant roles of lncRNAs in ovarian carcinogenesis and progression. For instance, LINC02776 has been shown to enhance OC drug resistance by increasing polyADP-ribosylation [14], while lncRNA KCNQ1OT1 promotes metastasis through regulation of the EIF2B5 promoter methylation [15]. However, the functions of numerous other lncRNAs in OC remain unexplored. Consequently, it is crucial to identify potential lncRNAs associated with OC metastasis.

Accumulating evidence suggests that lncRNAs can regulate mRNA stability, with RNA-binding proteins playing a crucial role in this process [16–18]. For instance, lncRNA SNHG16 interacts with EIF4A3 and enhances RhoU mRNA stability, thereby promoting the progression of esophageal squamous cell carcinoma [19]. Similarly, lncRNA PTTG3P promotes tumor growth and metastasis in NSCLC through binding to ILF3 to stabilize mRNA and establish a positive feedback loop with E2F1 [20]. On these accounts, the regulation of lncRNA-mediated mRNA can crucially affect cancer progression.

Axis inhibition protein 2 (AXIN2), a scaffold protein comprising multiple structural domains, operates by interacting with key molecules in the Wnt/β-catenin signaling pathway, namely glycogen synthase kinase-3β (GSK-3β) and β-catenin, leading to its aberrant activation. Despite extensive studies reporting the anti-tumor activity of AXIN2 in varying cancers, there are also studies indicating that it may also demonstrate oncogenic properties in colorectal, liver, and gastric cancers [21,22]. Considering these contrasting roles, the specific mechanisms of AXIN2 in tumorigenesis, especially in OC, remain ambiguous and warrant further investigation.

This study aims to probe into the biological function of CYMP-AS1 as well as its participation in OC occurrence and development. *In vivo* and *in vitro* experiments focused on analyzing the CYMP-AS1 expression in OC, evaluating its regulatory effect on OC cell proliferation, migration, invasion and EMT, and deeply explaining its potential molecular mechanism.

## 2 Materials and Methods

### 2.1 Patient and Clinical Samples

20 normal ovarian epithelial and 20 OC tissue specimens from the Second Hospital of Harbin Medical University (Harbin, Heilongjiang, China) underwent liquid nitrogen freezing immediately after excision and were stored at −80°C for the subsequent RNA extraction. This study acquired the approval of the Ethics Committee of the Hospital (KY2020-060) and followed the Declaration of Helsinki. All participants had given their written informed consent.

### 2.2 Cell Culture

The human OC cell lines, SKOV3, A2780, and OVCAR-3, along with the normal ovarian epithelial cell line, IOSE-80, came from the Shanghai Institute of Biochemistry and Cell Biology, Chinese Academy of Sciences. The A2780, OVCA-3, and IOSE-80 cells underwent culture in RPMI 1640 medium (Gibco, 11875093, Grand Island, NY, USA) with 100 U/mL antibiotics (Gibco, 15140148) and 10% FBS (Gibco, 10091148). SKOV3 cells were maintained in McCoy’s 5A medium (Pricella, PM150710, Wuhan, China) containing 10% FBS and 100 U/mL of penicillin. All cell lines underwent a certain period of incubation at 37°C in a humidified atmosphere with 5% CO_2_. All cells underwent STR profiling and were verified to be free of mycoplasma contamination.

### 2.3 Cell Transfection

Guangzhou RiboBio (Guangzhou, China) took charge of designing and synthesizing specific small interfering RNAs (siRNAs) targeting CYMP-AS1 (si-CYMP-AS1#1/#2) and hnRNPM (si-hnRNPM#1/#2), along with a negative control (si-NC). Table S1 lists the siRNA sequences. The X-tremeGene reagent (Roche, 06366236001, Basel, Switzerland) was employed for transfecting ovarian cells (SKOV3, A2780) with siRNAs. For *in vitro* transfection experiments, pcDNA3.1 (GenePharm, Shanghai, China) was used to construct pcDNA3.1/CYMP-AS1 and pcDNA3.1/hnRNPM, with an empty vector serving as a control. Plasmid transfection relied on Lipofectamine^®^ 2000 reagent (Invitrogen, 11668019, Carlsbad, CA, USA). After transfection in A2780 and SKOV3 cells, ovarian cells underwent 48 or 72 h of culture, after which RNA or proteins were extracted.

### 2.4 Fluorescence In Situ Hybridization (FISH)

Synthesis of Cy3-labeled CYMP-AS1 hybrid probes was conducted by Shanghai GenePharma (Shanghai, China). Fluorescent signals in transfected A2780 and SKOV3 cells were detected using a FISH kit (Genema, G0101, Suzhou, China) following the manufacturer’s protocol. DAPI (Beyotime, C1002, Shanghai, China) at 0.5–1 μg/mL served for nuclear staining with relevant images captured and analyzed using confocal microscopy (Olympus Corporation, FV1200, Tokyo, Japan).

### 2.5 RNA Extraction and Quantitative Real-Time PCR (RT-qPCR)

A specific kit was adopted for extracting total RNA from transfected ovarian cells and tissues using the TRIzol^®^ reagent (Invitrogen, 15596026, Carlsbad, CA, USA) as per the manufacturer’s protocol. cDNA was obtained via a reverse transcription (RT) Kit (Beyotime, D7168M, Shanghai, China). qPCR was conducted by virtue of SYBR Green MasterMix (Takara Bio, RR420A, Otsu, Japan) in a specific thermal cycling order: initial denaturation at 95°C for 1 min, 40 cycles of 95°C for 15 s and 52°C for 15 s. Table S2 lists the primer sequences. All RT-qPCR experiments were repeated three times, with expression data tested by virtue of the 2^−ΔΔCq^ method.

### 2.6 Protein Extraction and Western Blotting

Cells and tissues underwent ultrasonic lysis using RIPA lysis buffer (Beyotime, P0013B, Shanghai, China) to extract total proteins. After SDS-PAGE separation as per the producer’s protocol, nuclear and cytoplasmic proteins were transferred to nitrocellulose membranes (Millipore, IPVH00010, Burlington, MA, USA). After 2 h of blockage in 5% skimmed milk at room temperature (RT), the membranes underwent one night of incubation at 4°C using primary antibodies: anti-E-cadherin (Proteintech, 20874, Wuhan, China, 1:1000 dilution), anti-vimentin (Proteintech, 10366, 1:1000 dilution), anti-Snail (Cell Signaling Technology, 3879, Danvers, MA, USA, 1:100 dilution), anti-hnRNPM (Proteintech, 11714, 1:1500 dilution), anti-AXIN2 (Proteintech, 20540, 1:500 dilution), anti-c-MYC (Proteintech, 10828, 1:1000 dilution), anti-β-catenin (Cell Signaling Technology, 4970, 1:1000 dilution), anti-cyclin D1 (Proteintech, 60186, 1:1000 dilution), and anti-β-actin (Proteintech, 66009, 1:5000 dilution). Following three times of washes in 0.1% TBS-Tween, the nitrocellulose membranes received 1 h of incubation using horseradish peroxidase-conjugated goat anti-mouse (Cell Signaling Technology, 7074) or anti-rabbit (Cell Signaling Technology, 7076) secondary antibodies (dilution ratio of 1:3000) at RT. An ECL kit (Beyotime, P0018AM) assisted in visualizing the immunoreactive bands. Image analysis relied on ImageJ software (v1.54f, National Institutes of Health, Bethesda, MD, USA).

### 2.7 Cell Counting Kit-8 (CCK-8) Assay

The CCK-8 assay (Beyotime, C0038, Shanghai, China) was used to evaluate the cell proliferation. Experimenters seeded SKOV3 and A2780 cells into 96-well plates (2 × 10^3^ cells/well), with each well added with 10 μL of CCK-8 solution at 0, 24, 48, and 72 h, according to the producer’s protocol. Subsequently, the cells underwent another 1 h of incubation at 37°C. A microplate reader (Bio-Rad Laboratories, Hercules, CA, USA) was used for measuring the OD value at 450 nm.

### 2.8 EdU Staining

Experimenters seeded the transfected OC cells into 24-well plates (2 × 10^4^ cells/well). The Click-iT EdU Cell Proliferation Assay Kit (Beyotime, C0071S, Shanghai, China) was used for assessing the cell proliferation. The EdU working solution was prepared as per the producer’s protocol and incubated with the cells. Then, cells underwent fixation treatment in 4% paraformaldehyde (PFA) before being treated with a permeabilization solution (PBS that contained 0.3% Triton X-100) at RT. Nuclei were counterstained with 0.5 μg/mL DAPI solution alongside the visualization of cells via an epifluorescence microscope (Olympus Corporation, IX73, Tokyo, Japan).

### 2.9 Wound-Healing Assay

Transfected SKOV3 at a density of 5 × 10^5^ and A2780 cells at 2 × 10^5^ were seeded into six-well plates to be consistently cultured until reaching 90% confluence. A sterile pipette tip was employed to create wounds on the cell monolayer. Wound widths were measured via an inverted microscope (Nikon, ECLIPSE TS100, Tokyo, Japan) at 0 and 24 h following wound formation. Percentage of wound closure (%) = (width on Day 0–width on Day 24)/width on Day 0 × 100.

### 2.10 Transwell Assay

A2780 and SKOV3 cells received migration and invasion assays using Costar Transwell chambers (pore size: 8 μm, Corning, 3422, Corning, NY, USA). For the invasion assay, experimenters precoated the upper chamber with Matrigel (BD Biosciences, 356234, Franklin Lakes, NJ, USA) before cell seeding. The upper and lower chambers were filled with 200 μL of serum-free culture medium that contained 5 × 10^4^ cells and 600 μL of complete medium that contained 10% FBS, respectively. With non-invasive cells being removed using cotton swabs, the migratory or invasive cells, after fixation in 4% PFA, underwent 0.1% crystal violet staining, and were then imaged via an inverted microscope (Nikon, ECLIPSE TS100, Tokyo, Japan).

### 2.11 RNA Pull-Down Assay

An RNA pull-down kit (BoxinBio, K887-50, Guangzhou, China) was adopted for the RNA pull-down assays using biotin-labeled CYMP-AS1 *in vitro*-transcribed RNA (Genema, Suzhou, China) as per the producer’s protocol. Cell lysates of A2780 and SKOV3 cells were incubated with biotinylated RNA, followed by the addition of streptavidin agarose beads and overnight incubation at RT. Subsequently, RNA-bound proteins were retrieved through elution, detected using silver staining (Beyotime, P0017S, Shanghai, China), and identified via mass spectrometry (Thermo Fisher Scientific, BRE72547, Waltham, MA, USA). An RNA probe without biotin labeling served as a negative control.

### 2.12 RNA Immunoprecipitation (RIP)

The RIP assay was conducted on A2780 and SKOV3 cells using a RIP Kit (BoxinBio, K891-50, Guangzhou, China). In brief, cells were lysed with buffer, DNA was removed, and the cell lysate supernatant was subjected to one night incubation with magnetic beads carrying anti-hnRNPM or anti-IgG antibodies (Invitrogen, PA5-27208, Carlsbad, CA, USA). Subsequently, proteins were eluted with purified RNA and analyzed using RT-qPCR. The RT-qPCR products were then subjected to agarose gel electrophoresis.

### 2.13 RNA Stability

SKOV3 and A2780 cells were seeded at 5 × 10^4^ into 24-well plates and transfected. Subsequently, following treatment with 2.5 μg/mL of actinomycin D (MedChemExpress, HY-17559, Monmouth Junction, NJ, USA), cells were incubated. Cell samples were collected at 0, 4, and 8 h after incubation. RT-qPCR served for the extraction and analysis of RNA. This experiment was conducted in triplicate to ensure reliability.

### 2.14 Xenograft Tumor Model

Twelve 4-week-old female BALB/c nude mice (Beijing Vital River Laboratory Animal Technology, Beijing, China) were housed under specific pathogen-free (SPF) conditions with regulated environmental parameters and provided with autoclaved food and water. To establish a subcutaneous tumor model, each mouse was administered 1 × 10^7^ SKOV3 cells by subcutaneous injection into the left axilla. The mice fell into experimental and control groups in a random manner, followed by injection with 10 nmol cholesterol-modified CYMP-AS1 or control siRNAs, respectively, which are specifically designed for animal model construction (RiboBio, Guangzhou, China). The siRNAs were injected intratumorally every 3 days after tumor formation, with tumor volume recorded every 4 days. 32 days later, the mice were administered an overdose of 150 mg/kg sodium pentobarbital by intraperitoneal injection, with tumor tissues being harvested and weighed. All tumors in mice were <15 mm in diameter. This study followed the ARRIVE guidelines (https://arriveguidelines.org).

### 2.15 Bioinformatics Analysis

A public RNA-sequencing dataset comprising 31 OC and 32 normal ovarian epithelial tissue samples (GSE40595) was analyzed from the Gene Expression Omnibus (GEO) datasets. The gene expression was examined by utilizing data from the National Center for Biotechnology Information (NCBI, https://www.ncbi.nlm.nih.gov/). The bioinformatics website (http://www.csbio.sjtu.edu.cn/bioinf/lncLocator/ (accessed on 29 January 2025)) was used for the subcellular localization prediction.

### 2.16 Statistical Analyses

Statistical analysis relied on SPSS software (Version 22.0, IBM Corp., Armonk, NY, USA). Data were in the format of mean ± SD, based on three independent replicates. The Student’s *t*-test served for evaluating the between-group difference, while ANOVA served for the analysis of multivariate statistics. Survival between groups was calculated via the Kaplan–Meier method [23], and genetic correlations were evaluated using Spearman’s correlation [24]. *p* < 0.05 denotes statistical significance.

## 3 Results

### 3.1 CYMP-AS1 Was Upregulated in OC

**Figure 1:**
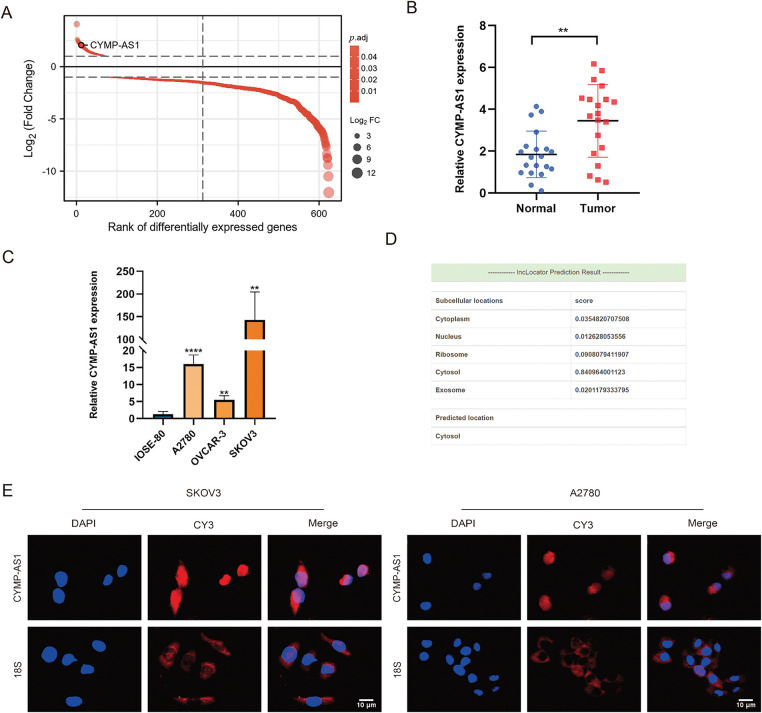
Upregulation of cymp-as1 in oc tissues and cells. (**A**) CYMP-AS1 expression comparison between OC and normal ovarian tissues utilizing GEO data; (**B**) RT-qPCR analysis of CYMP-AS1 expression in OC and normal ovarian tissues; (**C**) RT-qPCR analysis of CYMP-AS1 expression in OC and normal ovarian cells; (**D**) CYMP-AS1 localization in subcellular space predicted by bioinformatics tools; (**E**) RNA-FISH detection of CYMP-AS1 (red) expression in SKOV3 and A2780. Nucleus and CYMP-AS1 are stained blue (DAPI) and red, respectively. **p < 0.01, ****p < 0.0001

Bioinformatics analysis showed that CYMP-AS1 emerged as one of the most variable lncRNAs detected between OC and normal ovarian epithelial tissues (Fig. 1A). Given that CYMP-AS1 had not been previously investigated in the context of OC, this study aimed to elucidate its expression pattern, localization, and other characteristics. CYMP-AS1 is situated on human chromosome 1p13.3 and has a molecular length of 2579 bp. To validate CYMP-AS1 expression in OC, 20 specimens of OC and normal ovarian epithelial tissues were collected, and CYMP-AS1 expression was analyzed in tissues and cells using RT-qPCR. According to the analysis results, OC tissues exhibited remarkably higher CYMP-AS1 expression versus normal ovarian epithelial tissues (Fig. 1B). The median CYMP-AS1 expression in OC tissues was taken into account to categorize OC patients into low and high CYMP-AS1 groups. High CYMP-AS1 expression exhibited an obvious relevance to distant metastasis, lymph node metastasis, and tumor TNM stage (Table S3). According to Kaplan-Meier survival analysis, the high CYMP-AS1 expression group exhibited shorter OS (Fig. S1). Subsequently, CYMP-AS1 expression was examined in three OC cell lines, revealing higher levels compared to IOSE-80 cells. Notably, its expression was the highest in A2780 and SKOV3 cells (Fig. 1C). Bioinformatics predictions showed that CYMP-AS1 is primarily distributed in the cytoplasm (Fig. 1D). FISH experiments confirmed this prediction, demonstrating that while CYMP-AS1 was detected in both the cytoplasm and nucleus, it was predominantly observed in the cytoplasm (Fig. 1E).

### 3.2 CYMP-AS1 Knockdown Prevented OC Cell Proliferation, Migration, Invasion, and EMT In Vitro

**Figure 2:**
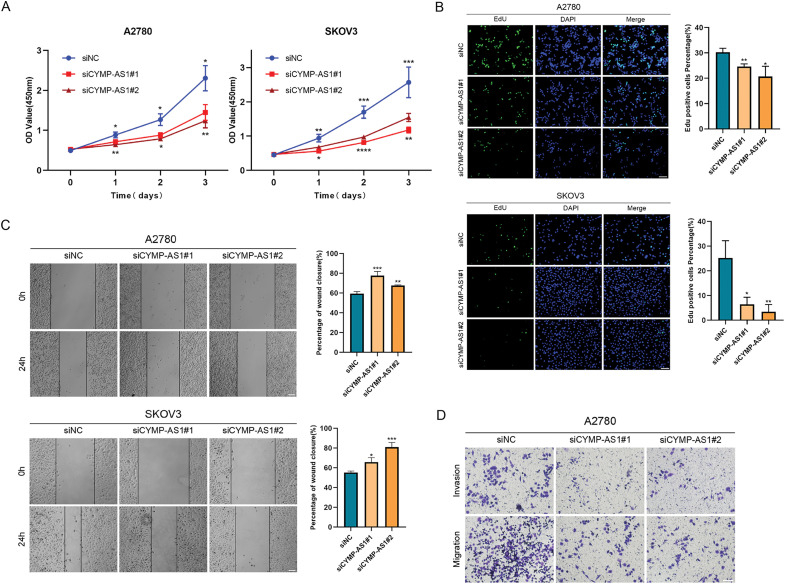

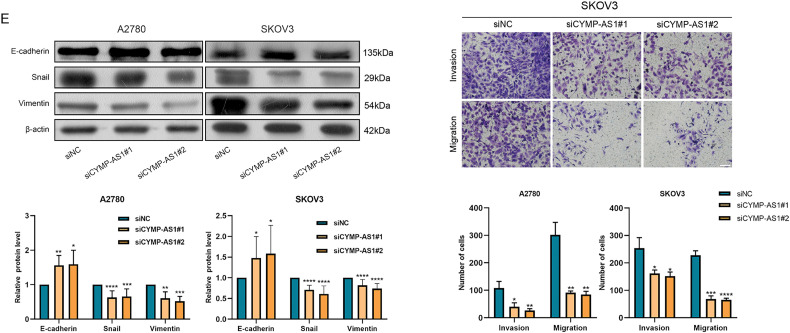
CYMP-AS1 knockdown inhibits OC cell progression. (**A,B**) Proliferative capacity of A2780 and SKOV3 cells following CYMP-AS1 knockdown using CCK-8 and EdU assays (scale bar, 50 μm); (**C,D**) Cell migration and invasion were evaluated in OC cells with or without CYMP-AS1 knockdown using Wound healing (scale bar, 200 μm) and Transwell (scale bar, 100 μm) assays; (**E**) CYMP-AS1 knockdown reduced EMT progression in SKOV3 and A2780 cells. *p < 0.05, **p < 0.01, ***p < 0.001, ****p < 0.0001

To investigate the function of CYMP-AS1 in OC development, SKOV3 and A2780 cells exhibiting high CYMP-AS1 expression were treated with two siRNAs. RT-qPCR confirmed successful CYMP-AS1 knockdown in both cell lines, enabling their use in subsequent experiments (Fig. S2A). A CCK-8 assay was conducted with regard to the participation of CYMP-AS1 in human OC cell proliferation, which indicated that CYMP-AS1 knockdown significantly reduced cellular proliferative capacity (Fig. 2A), corroborating EdU assay findings and, further substantiating the role of CYMP-AS1 in promoting A2780 and SKOV3 cell proliferation (Fig. 2B). Additionally, according to wound healing together with Transwell assays OC cells exhibited weaker migration and invasion following CYMP-AS1 silencing (Fig. 2C,D). EMT markers in OC cells were subjected to Western blotting analysis, demonstrating higher E-cadherin levels and lower Snail and vimentin levels after CYMP-AS1 knockdown (Fig. 2E). These findings suggest that CYMP-AS1 knockdown restricts OC cells from proliferating, migrating and invading, and weakens EMT progression *in vitro*.

### 3.3 CYMP-AS1 Overexpression Promoted OC Cell Proliferation, Migration, Invasion, and EMT In Vitro

**Figure 3:**
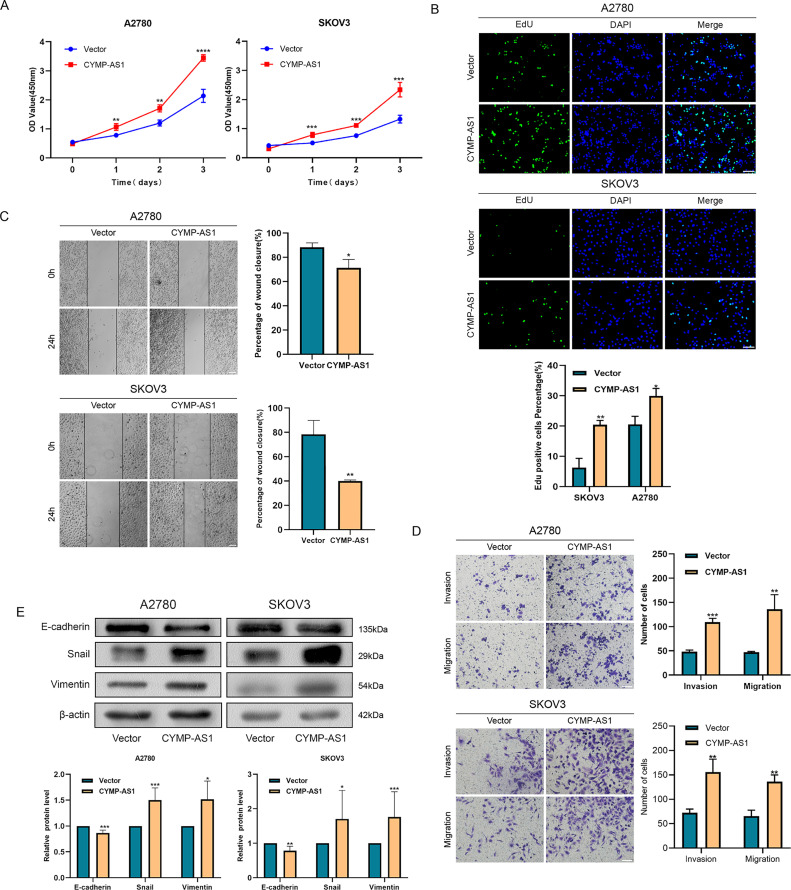
Overexpression of CYMP-AS1 promotes OC cell progression. (**A**) CCK-8 assay assessed SKOV3 and A2780 cell proliferation with CYMP-AS1 overexpression; (**B**) The proliferative capacity of OC cells with CYMP-AS1 overexpression was evaluated using EdU (scale bar, 50 μm); (**C**) Wound healing assay analyzed the cell migration and invasion abilities of OC cells with CYMP-AS1 overexpression (scale bar, 200 μm); (**D**) Transwell assay was conducted on SKOV3 and A2780 cells with CYMP-AS1 overexpression to investigate cell migration and invasion capabilities (scale bar, 100 μm); (**E**) Western blot analysis showing the enhancement of EMT progression in SKOV3 and A2780 cells by CYMP-AS1 overexpression. *p < 0.05, **p < 0.01, ***p < 0.001, ****p < 0.0001

The lncRNA was introduced into two OC cell lines using overexpression vectors, aiming at more deeply elucidating the role of CYMP-AS1 in OC. RT-qPCR subsequently verified the overexpression efficiency, confirming their suitability for subsequent experiments (Fig. S2B). According to CCK-8 and EdU assays, CYMP-AS1 overexpression contributed to an obvious improvement in OC cell proliferative capacity (Fig. 3A,B). According to Wound healing and Transwell assays, CYMP-AS1 overexpression markedly increased OC cells migration and invasion (Figs. 3C,D). Furthermore, according to western blotting analysis, CYMP-AS1 overexpression in OC cells lowered the E-cadherin expression and elevated the Snail and Vimentin expression (Fig. 3E). Collectively, these findings suggest that CYMP-AS1 overexpression facilitates OC cells to proliferate, migrate and invade, as well as enhances EMT progression.

### 3.4 CYMP-AS1 Promoted OC Progression In Vivo

**Figure 4:**
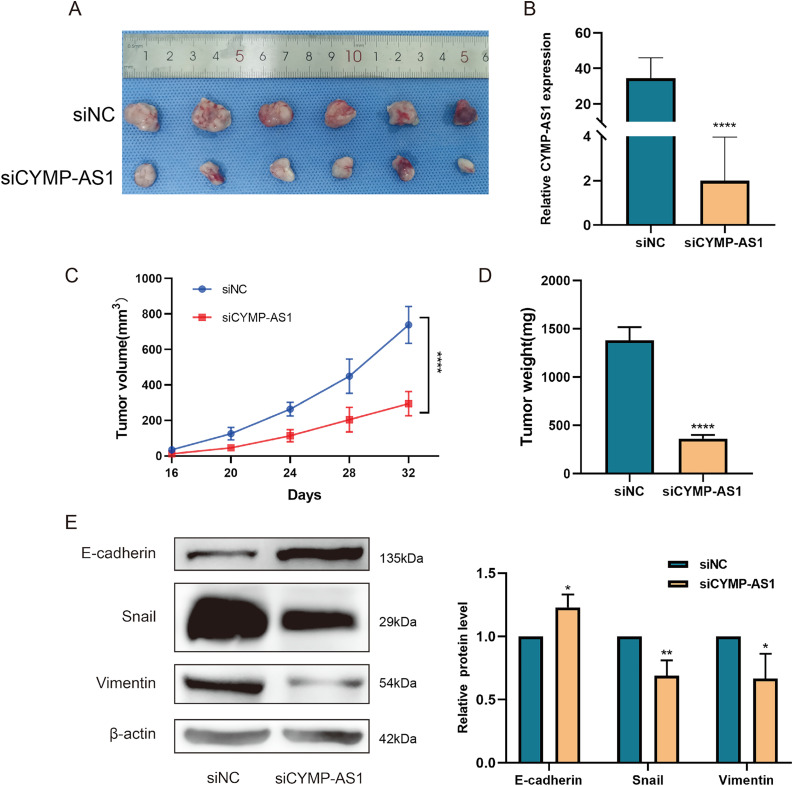
CYMP-AS1 knockdown inhibits tumor growth in vivo. (**A**) Representative images of OC tumors in BALB/c nude mice; (**B**) RT-qPCR analysis of CYMP-AS1 expression in tumor tissues; (**C**) Measurement of tumor volume every 4 days; (**D**) Comparison of tumor weights between the CYMP-AS1 knockdown group and the control group; (**E**) Expression level of E-cadherin, Snail, and vimentin in tumor xenograft biopsies via Western blot analysis. *p < 0.05; **p < 0.01; ****p < 0.0001

With the aim of deeply revealing the role of lncRNA CYMP-AS1 in OC tumorigenesis, we used SKOV3 cells to construct a subcutaneous transplant tumor model. Compared to the control group, tumor size significantly decreased after CYMP-AS1 knockdown (Fig. 4A). The results of RT-qPCR experiments confirmed a substantial reduction in CYMP-AS1 expression after knockdown, validating the effectiveness of the animal experimental model (Fig. 4B). CYMP-AS1 knockdown remarkably decreased the tumor tissue volume and weight versus the control group (Fig. 4C,D). Collectively, CYMP-AS1 likely promotes OC cell growth *in vivo*. Additionally, western blotting served to assess E-cadherin, Snail, and vimentin expressions in mouse tumor tissues. The CYMP-AS1 knockdown group exhibited higher E-cadherin expression and lower Snail and vimentin expressions versus the control group (Fig. 4E). On these accounts, CYMP-AS1 significantly affects the OC progression *in vivo*.

### 3.5 CYMP-AS1 Specifically Binds to hnRNPM and Alters Its Subcellular Localization

**Figure 5:**
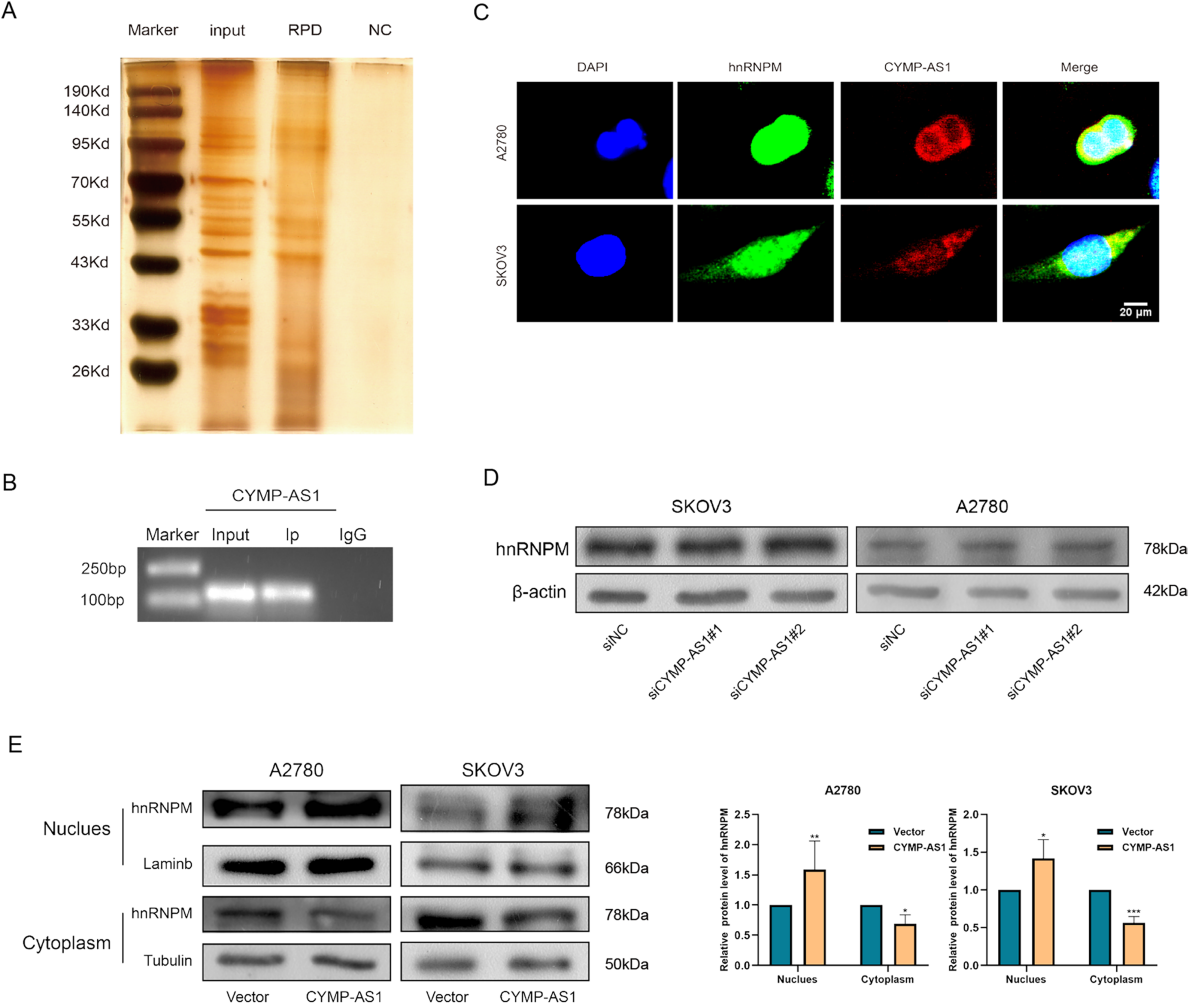

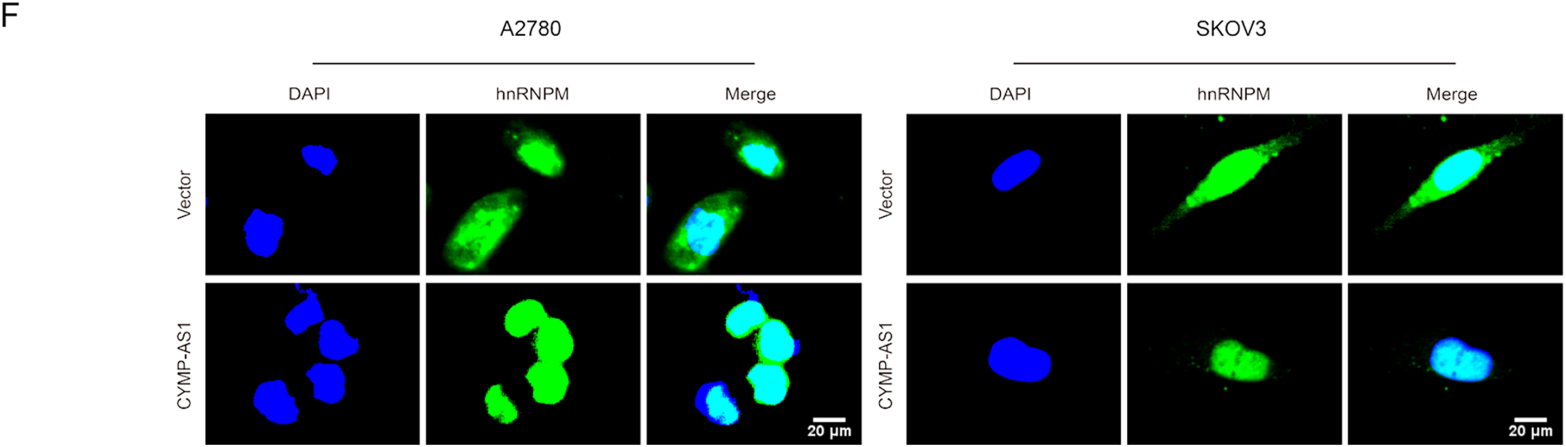
HnRNPM directly binds to CYMP-AS1. (**A**) RNA pull-down assay and silver staining experiments to identify CYMP-AS1-protein complexes; (**B**) RNA immunoprecipitation assay demonstrating CYMP-AS1 enrichment in the hnRNPM protein; (**C**) IF-FISH analysis to compare the relative expression of CYMP-AS1 and hnRNPM; (**D**) Western blotting analysis of hnRNPM expression levels in CYMP-AS1 knockdown OC cells. (**E**) Western blot analysis investigating the impact of CYMP-AS1 on hnRNPM protein levels in the nuclear and cytoplasm; (**F**) The subcellular localization of hnRNPM in CYMP-AS1 overexpressed OC cells was investigated by immunofluorescence assay. *p < 0.05; **p < 0.01; ***p < 0.001

According to recent research, lncRNAs can function by binding to proteins [25]. To identify the possible protein-binding partners of CYMP-AS1 in OC cells, we conducted RNA pull-down experiments, mass spectrometry and silver staining in succession. Our analysis identified hnRNPM as one of the most abundant proteins interacting with CYMP-AS1 (Fig. 5A). The hnRNPM protein, a component of the spliceosome machinery, has been implicated in regulating tumor cell invasion, metastasis, and EMT progression [26]. Consequently, hnRNPM was selected for future mechanistic studies. The interaction between hnRNPM and CYMP-AS1 in OC cells was further confirmed using RIP experiments (Fig. 5B). Moreover, immunofluorescence analysis demonstrated colocalization of hnRNPM and CYMP-AS1 (Fig. 5C). We then investigated whether CYMP-AS1 regulates hnRNPM expression. Western blotting revealed that CYMP-AS1 overexpression did not change the total protein expression level of hnRNPM in SKOV3 and A2780 cells (Fig. 5D). Previous studies have reported that some hnRNPs shuttle between the nucleus and cytoplasm [27], facilitating their binding to target RNAs. Therefore, we examined the altered distribution of hnRNPM in OC cells with high CYMP-AS1 expression. Western blotting and immunofluorescence results indicated that CYMP-AS1 overexpression reduced hnRNPM levels in the cytoplasm, while increasing its expression in the nucleus (Fig. 5E,F). Collectively, these findings demonstrate that CYMP-AS1 specifically binds to hnRNPM and facilitates its translocate from the cytoplasm to the nucleus.

### 3.6 CYMP-AS1 Functions in OC by Promoting the Intracellular Translocation of hnRNPM

**Figure 6:**
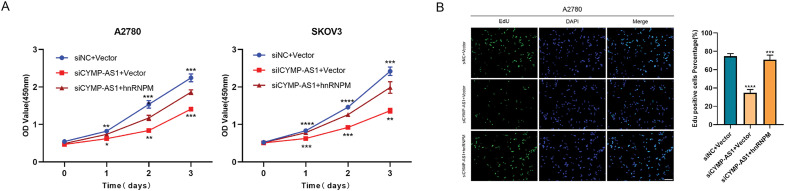

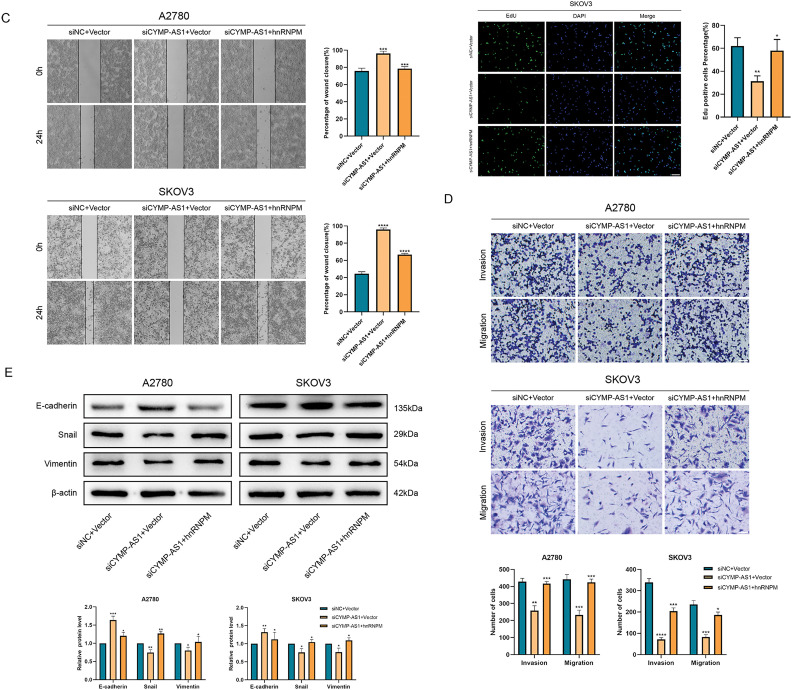
CYMP-AS1 promoted OC cell progression through the intracellular translocation of hnRNPM. (**A**) Cell viability assessment via a CCK-8 assay; (**B**) Cell proliferation capacity evaluation via an EdU assay (scale bar, 50 μm); (**C,D**) Cell migration and invasion were analyzed using wound healing (scale bar, 200 μm) and Transwell assays (scale bar, 100 μm); (**E**) Expression detection of EMT-related proteins via western blot analysis. *p < 0.05, **p < 0.01, ***p < 0.001, ****p < 0.0001

To elucidate whether hnRNPM functions as a downstream mediator of CYMP-AS1’s role in OC cell proliferation and metastasis, rescue experiments were conducted. According to the CCK-8 and EdU assays, co-transfection with an hnRNPM-overexpression plasmid containing a nuclear localization signal sequence reversed the reduced proliferation of OC cells caused by CYMP-AS1 knockdown (Fig. 6A,B). Similarly, according to wound healing and Transwell assays. OC cells exhibited worse migratory and invasive capabilities resulting from CYMP-AS1 knockdown, and such impact could be mitigated by intracellular translocation of hnRNPM (Fig. 6C,D). Moreover, western blotting analysis indicated that intracellular translocation of hnRNPM reversed the inhibitory mechanism of CYMP-AS1 downregulation against EMT progression, as evidenced by changes in E-cadherin, vimentin and Snail protein levels (Fig. 6E).

### 3.7 CYMP-AS1 and hnRNPM Interacted to Activate Wnt**/β**-Catenin Signaling

**Figure 7:**
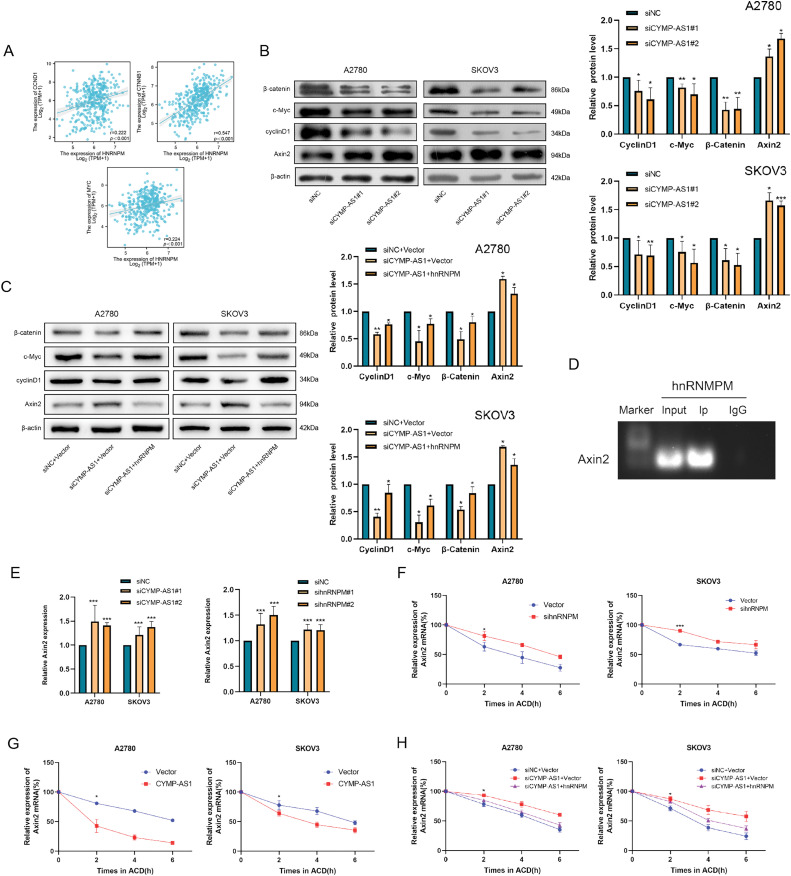
CYMP-AS1 reduces AXIN2 mRNA stability by interacting with hnRNPM. (**A**) Correlation analysis of CYMP-AS1 and Wnt/β-catenin signaling proteins; (**B**) Expressions of Wnt/β-catenin signaling proteins in OC cells with CYMP-AS1 knockdown; (**C**) CYMP-AS1 regulates the expression of proteins through hnRNPM in the nucleus; (**D**) The interaction of hnRNPM and AXIN2 mRNA was detected by RIP assay; (**E**) RT-qPCR analyses verified the AXIN2 mRNA expression in OC cells; (**F**) AXIN2 mRNA stability was increased by hnRNPM knockdown versus the control group; (**G**) AXIN2 mRNA stability was decreased by CYMP-AS1overexpression versus the control group; (**H**) CYMP-AS1 decreased AXIN2 mRNA stabilization through hnRNPM in the nucleus. *p < 0.05, **p < 0.01, ***p < 0.001

HnRNPM was found to exhibit a positive relevance to the expression of pathway-related proteins using Spearman’s correlation coefficient with The Cancer Genome Atlas (TCGA)-High-Grade Serous (HGS)-OC data (Fig. 7A). Western blot analysis of OC cells with CYMP-AS1 knockdown revealed downregulation of pathway-related proteins, including cyclin D1, c-MYC and β-catenin, but the expression of the pathway regulator AXIN2 was upregulated (Fig. 7B). Conversely, translocation of hnRNPM into the nucleus partially reversed the alterations in pathway protein expression caused by CYMP-AS1 knockdown (Fig. 7C). These findings suggest that CYMP-AS1 may interact with hnRNPM to regulate target genes specific to the Wnt/β-catenin signaling pathway.

### 3.8 Interaction between CYMP-AS1 and hnRNPM Suppressed the Stability of AXIN2 mRNA

The study further elucidated the potential mechanism allowing CYMP-AS1 to activate the Wnt/β-catenin signaling pathway through interactions with hnRNPM. Given that hnRNPs have been reported to influence mRNA stability, it was hypothesized that they might act on the mRNA of the pathway regulator AXIN2. To test this hypothesis, RIP experiments were conducted, demonstrating that hnRNPM binds to AXIN2 mRNA in OC cells (Fig. 7D). Additionally, knockdown of both CYMP-AS1 and hnRNPM resulted in increased AXIN2 mRNA expression levels (Fig. 7E). Actinomycin D treatment inhibited the ongoing transcription of cells and AXIN2 mRNA expression levels were assessed using RT-qPCR. Accordingly, hnRNPM knockdown delayed AXIN2 mRNA degradation in SKOV3 and A2780 cells (Fig. 7F), indicative of the role of hnRNPM in reducing AXIN2 mRNA stability. Furthermore, CYMP-AS1 overexpression decreased AXIN2 mRNA stability (Fig. 7G). However, the translocation of hnRNPM into the nucleus counteracted the reduction in mRNA stability caused by CYMP-AS1 underexpression (Fig. 7H). These findings suggest that hnRNPM and CYMP-AS1 synergistically reduce AXIN2 mRNA stability, thereby modulating the process that the Wnt/β-catenin signaling pathway is transactivated.

## 4 Discussion

OC often results in a poor prognosis owing to its aggressive and metastatic nature [28]. EMT is a crucial cellular process significantly affecting the tumor cell invasion and metastasis [29–31]; however, the mechanisms governing its regulation remain largely unexplored. According to recent studies, lncRNAs significantly regulate tumor development [32–34]. In our study, CYMP-AS1 promoted OC cells to be largely proliferate, migrated and invaded, and intensified the EMT progression. CYMP-AS1 exerted pro-cancer effects by binding to hnRNPM, reducing the mRNA stability of AXIN2, and modulating the Wnt/β-catenin signaling pathway, ultimately accelerating the OC progression.

Although lncRNAs crucially impact cancer progression, their involvement in OC remains to be fully elucidated. To identify lncRNAs associated with the advancement of progressive metastasis in OC, the study analyzed the expression profiles pertaining to OC and normal ovarian epithelium tissues from public databases. CYMP-AS1 emerged as one of the lncRNAs with the highest differential expression profile. To further validate this finding, this study examined the expression of CYMP-AS1 in clinical specimens. Collectively, epithelial plasmacytoid OC tissues exhibited considerably higher CYMP-AS1 expression versus normal ovarian epithelial tissues. On these accounts, CYMP-AS1 may be a biomarker and potential prognostic predictor for OC.

Elevated CYMP-AS1 expression contributed to an obvious enhancement in SKOV3 and A2780 cells in terms of their proliferation, migration, and invasion, while its suppression diminished these effects. EMT is a well-established process enabling cancer cells to transform from an epithelial phenotype to a mesenchymal phenotype [35–37]. According to Western blotting analysis, CYMP-AS1 overexpression increased Snail and vimentin expression while decreasing E-cadherin levels. Conversely, CYMP-AS1 silencing exhibited the opposite trend. *In vivo* experiments corroborated these findings. Collectively, CYMP-AS1 overexpression promotes OC cells to be more largely proliferate, migrate and invade and induces EMT, ultimately benefiting the OC progression.

The subcellular localization of RNA significantly influences various cellular activities [38,39], suggesting that the distribution of lncRNAs, as functional units, substantially affects their roles. In the nucleus, lncRNAs work on modulating transcription by interacting with chromatin and remodeling machinery, while in the cytoplasm, lncRNAs primarily mediate signal transduction pathways, translation programs, and post-transcriptional regulation of gene expression. According to recent studies, cytoplasmic lncRNAs construct complex protein networks involved in signal transduction as scaffolds [25,27]. This study found that CYMP-AS1 is distributed in OC cells’ nucleus and cytoplasm, with greater abundance in the cytoplasm, suggesting that CYMP-AS1 may primarily function by binding to specific proteins. RNA pull-down and RIP experiments confirmed the direct binding of CYMP-AS1 to hnRNPM. The hnRNP family comprises key variable splice regulators involved in mRNA maturation and translocation, recognizing and regulating various biological processes related to RNA function and metabolism. Additionally, hnRNPs pivotally impact the development of malignant tumors [40,41]. HnRNPs can shuttle between the nucleus and cytoplasm, consequently affecting biological processes [42,43]. The hnRNPM contains three RNA recognition structural domains that regulate RNA alternative splicing and has recently been shown to induce EMT in cancer and contribute to the maintenance of the mesenchymal phenotype [44,45]. This study’s experiments demonstrated that upregulated expression of CYMP-AS1 led to the relocation of hnRNPM from the cytoplasm to the nucleus, and hnRNPM knockdown rescued proliferation, migration, invasion, and EMT enhancement caused by CYMP-AS1 overexpression. Bioinformatics analysis revealed that overexpression of CYMP-AS1 alters the expression of Wnt/β-catenin signaling pathway genes, whose activation has been reported to lead to EMT and progression of malignant tumors. Examination of altered protein-expression levels in the pathway after CYMP-AS1 knockdown showed downregulation of cyclin D1, c-MYC, and β-catenin expression. These findings further confirm hnRNPM, as a downstream mechanism by which CYMP-AS1 promotes OC EMT progression via the Wnt/β-catenin pathway.

Further investigation into the mechanism that enables CYMP-AS1 to activate the Wnt/β-catenin pathway revealed its association with AXIN2 mRNA stability. AXIN is a scaffold protein that can connect GSK-3β, CK1α, APC, and β-catenin, driving β-catenin phosphorylation and degradation. Axin2 crucially participates in cancer development through modulating the signaling pathway. While in some studies, AXIN2 may have oncogenic properties, its role as a Wnt/β-catenin inhibitor and tumor suppressor is more widely recognized. Specifically, in colorectal cancer, ALKBH5 exhibits an immunosuppressive activity by targeting AXIN2 [46]. In gastric cancer, LPAR2 reduces AXIN2 expression, activating the Wnt/β-catenin pathway and promoting cancer cells to be proliferated, migrated and invaded [47]. AXIN2 mRNA stability has been reported to be regulated through various mechanisms [48]. This study found that upregulated CYMP-AS1 expression led to hnRNPM relocation from the cytoplasm to the nucleus, promoting binding between hnRNPM and AXIN2 mRNA, thereby reducing AXIN2 mRNA stability. This interaction ultimately activates the Wnt/β-catenin signaling pathway.

This study has some limitations. First, the complete regulatory mechanisms through which CYMP-AS1 modulates the Wnt/β-catenin signaling axis remain to be fully elucidated. Furthermore, the clinical relevance and therapeutic potential of the CYMP-AS1/hnRNPM/AXIN2 pathway require rigorous validation through well-designed clinical studies to assess its applicability in real-world settings.

## 5 Conclusions

This study provides evidence that lncRNA CYMP-AS1 promotes the progression of OC. Mechanistic analyses reveal that CYMP-AS1 binds to hnRNPM, resulting in its intracellular translocation. This interaction reduces the stability of AXIN2 mRNA, subsequently activating the Wnt/β-catenin pathway, and benefits OC cell proliferation, invasion, infiltration, and EMT. Collectively, CYMP-AS1 is possibly a potential target in treating OC.

## Supplementary Materials

Figure S1 Overall survival of patients with ovarian cancer based on high or low CYMP-AS1 expression.

Figure S2 Transfection efficiency of CYMP-AS1 in OC cells. **(A)** Knockdown efficiency of CYMP-AS1 in SKOV3 and A2780 cells. **(B)** Overexpression efficiency of CYMP-AS1 in SKOV3 and A2780 cells. *p<0.05, **p<0.01, ****p<0.0001.

## Data Availability

The datasets used and analyzed during the current study are available from the corresponding author upon reasonable request.

## Supplementary Materials

The supplementary material is available online at https://www.techscience.com/doi/10.32604/or.2025.064367/s1.

## Abbreviations

AXIN2: Axis inhibition protein 2
CCK-8: Cell counting kit 8
Edu: 5-ethynyl-2^′^-deoxyuridine
EMT: Epithelial mesenchymal transition
FISH: Fluorescence in situ hybridization
lncRNA: Long non-coding RNA
OC: Ovarian cancer
RIP: RNA immunoprecipitation
