# Supplementary figures and images for: CYMP-AS1 Promotes Ovarian Cancer Progression by Enhancing the Intracellular Translocation of hnRNPM and Reducing the Stability of AXIN2 mRNA
Source: Oncol Res. 2025 Jul 18;33(8):2141–59. doi: 10.32604/or.2025.064367 (PMC12308254; doi:10.32604/or.2025.064367)

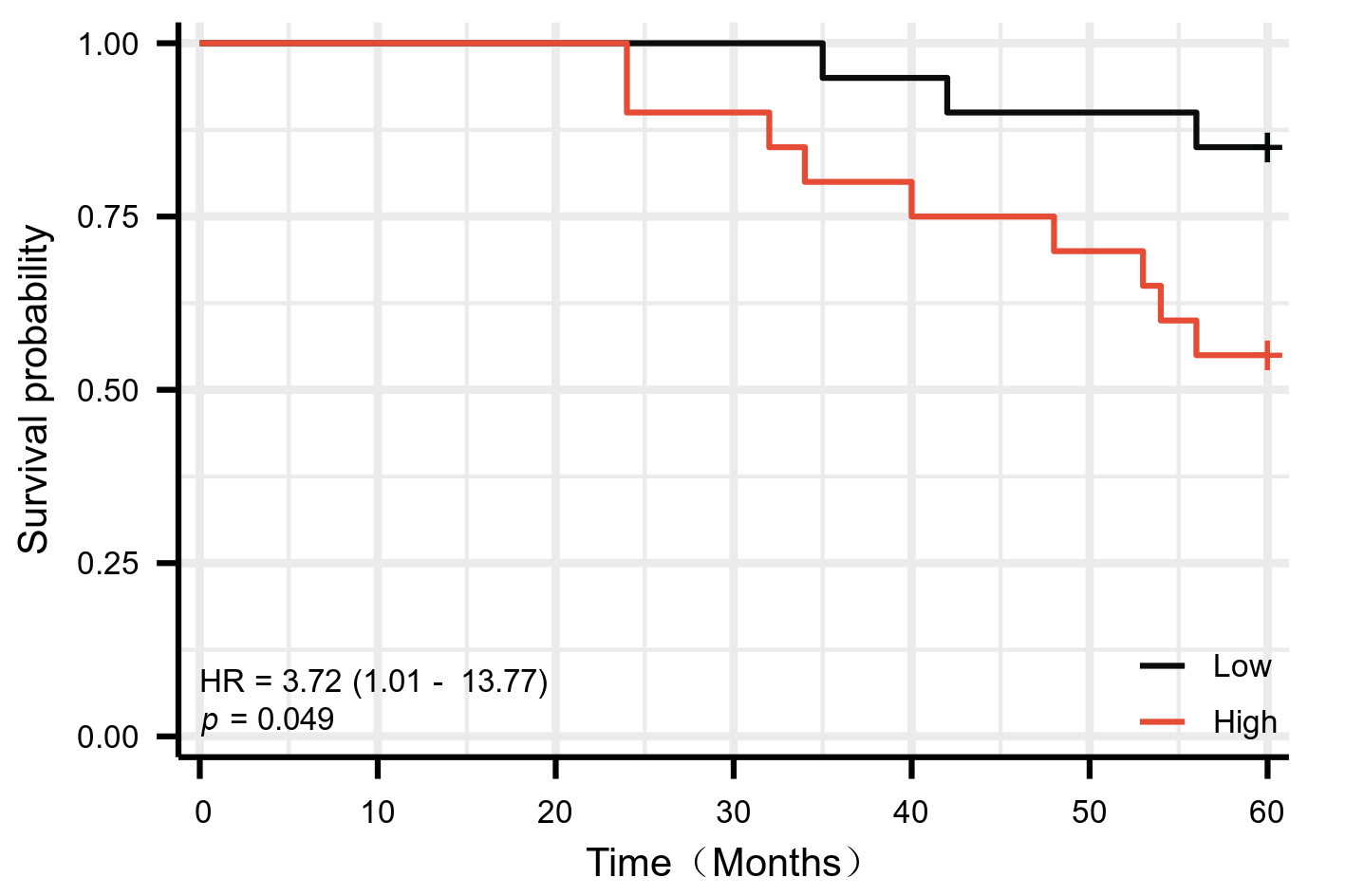

Supplement: Figure S1 [file OncolRes-33-64367-s001.tif]
