# Supplementary material for: CYMP-AS1 Promotes Ovarian Cancer Progression by Enhancing the Intracellular Translocation of hnRNPM and Reducing the Stability of AXIN2 mRNA
Source: Oncol Res. 2025 Jul 18;33(8):2141–59. doi: 10.32604/or.2025.064367 (PMC12308254; doi:10.32604/or.2025.064367)
Supplement: Supplementary file 3 [file OncolRes-33-64367-s003.docx]

**Supplementary Materials**

**Table S1:** siRNA sequences used in this study.

| **Name** | **Sequence** |
| --- | --- |
| siCYMP-AS1#1 | GCCTGAGATCTGGTTTCAA |
| siCYMP-AS1#2 | GCAGCTGGTCTTGTTCATA |
| siHNRNPM#1 | GGTGTGGCGTGGTTAAGTT |
| siHNRNPM#2 | GAGAGATTGACGTTCGAAT |

**Table S2.** Primer sequences of **RT-qPCR** used in this study.

| **Gene name** | | **Sequence (5'-3')** |
| --- | --- | --- |
| CYMP-AS1 | Forward | CCACTCCTCCCTCAGCAAAG |
|  | Reverse | GAAGCAGACAGCACACCATTAC |
| HNRNPM | Forward | CATGGCCTTGGTGGTATTGG |
|  | Reverse | TTCCTGCGGGACCTATGTTT |
| AXIN2 | Forward | TCAAGACGGTGCTTACCTGT |
|  | Reverse | TTGCAGGCAAACCAGAAGTC |
| β-actin | Forward | TCGTGCGTGACATTAAGGAGAAG |
|  | Reverse | GTTGAAGGTAGTTTCGTGGATGC |

**Table S3.** Association of CYMP-AS1 expression with clinicopathological features of the OC.

| **Characteristic** | | **High** | **Low** | ***p* value** |
| --- | --- | --- | --- | --- |
| n | | 20 | 20 |  |
| T stage, n (%) | |  |  | 0.0005 |
|  | T1 | 16 | 4 |  |
|  | T2 | 3 | 7 |  |
|  | T3 | 1 | 9 |  |
| N stage, n (%) | |  |  | 0.0004 |
|  | N0 | 16 | 5 |  |
|  | N1 | 4 | 15 |  |
| M stage, n (%) | |  |  | 0.0015 |
|  | M0 | 16 | 6 |  |
|  | M1 | 4 | 14 |  |
| Tumor size (cm), n (%) | |  |  | 0.1840 |
|  | <2 | 19 | 15 |  |
|  | ≥2 | 1 | 5 |  |
| Lymph node metastasis, n (%) | |  |  | 0.0005 |
|  | Negative | 16 | 5 |  |
|  | Positive | 4 | 15 |  |
| Distant metastasis, n (%) | |  |  | 0.0001 |
|  | Negative | 16 | 4 |  |
|  | Positive | 4 | 16 (40%) |  |

**
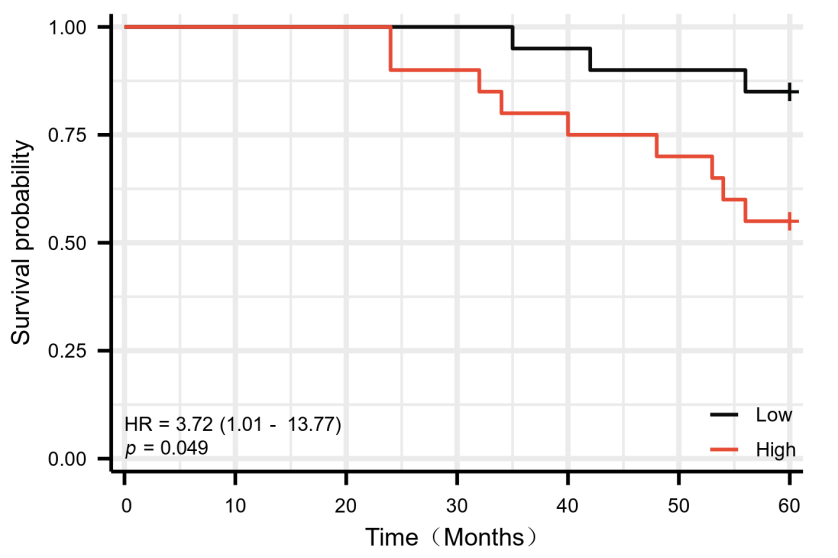
**

**Figure S1.** Overall survival of patients with ovarian cancer based on high or low CYMP-AS1 expression.


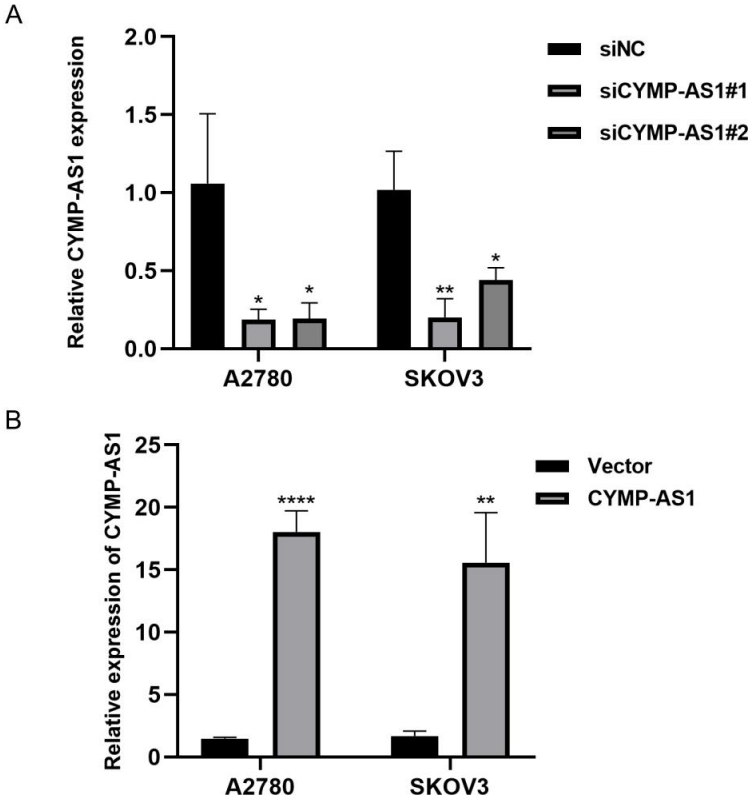


**Figure S2.** Transfection efficiency of CYMP-AS1 in OC cells. **(A)** Knockdown efficiency of CYMP-AS1 in SKOV3 and A2780 cells. **(B)** Overexpression efficiency of CYMP-AS1 in SKOV3 and A2780 cells. **p*<0.05, ***p*<0.01, *****p*<0.0001.
